# Giant-Cell Tumor of the Distal Ulna Treated by Wide Resection and Ulnar Support Reconstruction: A Case Report

**DOI:** 10.1155/2010/871278

**Published:** 2010-06-13

**Authors:** Akio Minami, Norimasa Iwasaki, Kinya Nishida, Makoto Motomiya, Katsuhisa Yamada, Daisuke Momma

**Affiliations:** Department of Orthopaedic Surgery, Hokkaido University Graduate School of Medicine, Sapporo 060-8638, Japan

## Abstract

Giant-cell tumor of bone occurred in the distal end of the ulna is extremely uncommon. A 23-year-old male had a giant-cell tumor occurred in the distal end of the ulna. After wide resection of the distal segment of the ulna including giant-cell tumor, ulnar components of the wrist joint were reconstructed with modified Sauvé-Kapandji procedure using the iliac bone graft, preserving the triangular fibrocartilage complex and ulnar collateral ligament in order to maintain ulnar support of the wrist, and the proximal stump of the resected ulna was stabilized by tenodesis using the extensor carpi ulnaris tendon. One year after operation, the patient's wrist was pain-free and had a full range of motion. Postoperative X-rays showed no abnormal findings including recurrence of the giant-cell tumor and ulnar translation of the entire carpus. The stability of the proximal stump of the distal ulna was also maintained.

## 1. Introduction

 Giant-cell tumor (GCT) of the bone is a rare, benign, and locally invasive tumor. It is accounting for about 3% to 5% of all primary bone tumors [1]. GCTs of the bone usually occur at the epiphysis of the long bone such as femur, tibia, humerus, and radius. GCTs occurred at the distal end of the ulna are extremely rare, accounting for 0.45% to 3.2% of all the cases of GCTs [2]. This paper described a young male with a GCT of the distal end of the ulna treated by a wide resection and ulnar support reconstruction of the wrist. 

## 2. Case Report

 A 23-year-old male, manual laborer, noticed a movemental pain and swelling around the ulnar head of the left wrist on January, 2008. Pain suddenly increased two months after the onset without any particular event. The patient was seen to a clinic on March, 2008. In there, the patient was informed that there was an abnormal shadow in the ulnar head of the left wrist. There was no history of any other swelling in the body, fever, and loss of weight. The patient was introduced and first seen in our hospital on May, 2008. 

 Physical examinations revealed that there was an oval swelling of 4 × 3 cm in the distal end of the ulna. There was no color change and redness on the overlying skin. The swelling was diffusely tender and uniformly elastically hard. There was no adherence of the skin to the under lying bone. The range of motion of the patient's left wrist was limited to 60° (contralateral side: 80°) in dorsiflexion and 50° (80°) in palmar flexion, 60° (90°) in pronation and 80° (90°) in supination. Moderate movemental pain was present at the extremes in all directions. The grip strength of his nondominant left wrist showed 27 kgf compared with 42 kgf of the unaffected dominant hand. 

**Figure 1:**
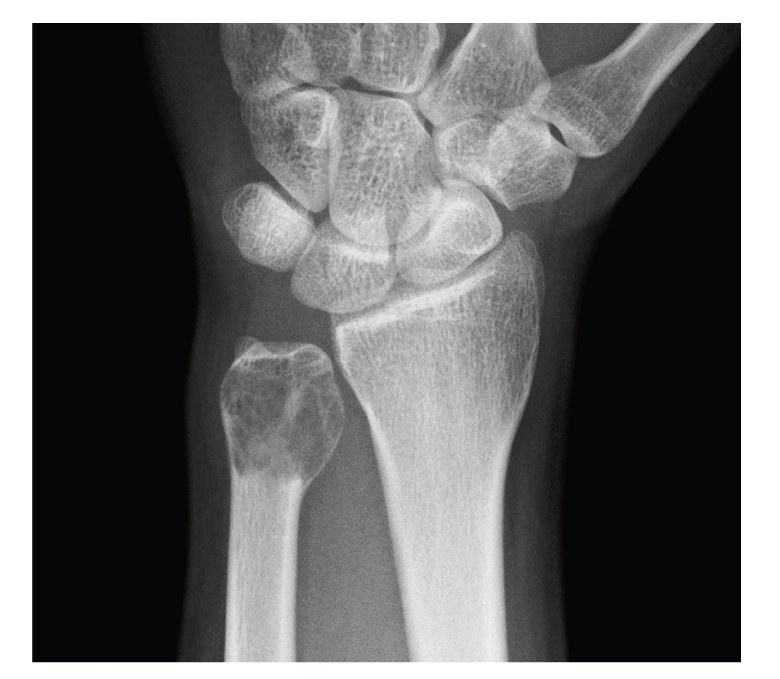
Preoperative plain X-ray showed an expansile, multilobular, and radiolucent lesion with a clear margin in the distal end of the left ulna.

**Figure 2:**
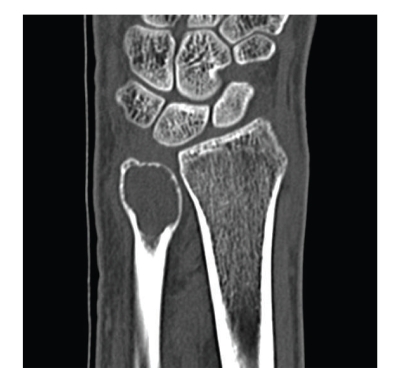
Computed tomogram showed thinning and protrusion of the cortex but no destruction of the cortex of the distal ulna.

 Blood examinations were within normal limits. Plain X-ray of the left ulna showed an expansile, multilobular, and radiolucent lesion with a clear margin, so-called soap-bubbled appearance lesion at the distal end with absence of periosteal reaction and incomplete fracture (Figure 1). Other X-rays including chest showed no abnormality. Computed tomograms showed thinning and protrusion of the cortex, but no destruction of the cortex of the distal ulna (Figure 2). Magnetic resonance image (MRI) showed a low intensity in T1 weighted image and a relatively high intensity in T2 weighted image. A clinical diagnosis of GCT was made. Therefore, open biopsy was performed to make an accurate diagnosis. Histological findings revealed that the tumor was consisted of mononuclear tumor cells with eosinophilic oval and short fusiform nucleus and osteoclastic multinuclear giant cells, indicating typical benign GCT of the bone. On the basis of clinical and radiographic evaluations, the lesion was graded as stage 3 (aggressive) as per the Enneking Staging system for benign bone tumors [3].

**Figure 3:**
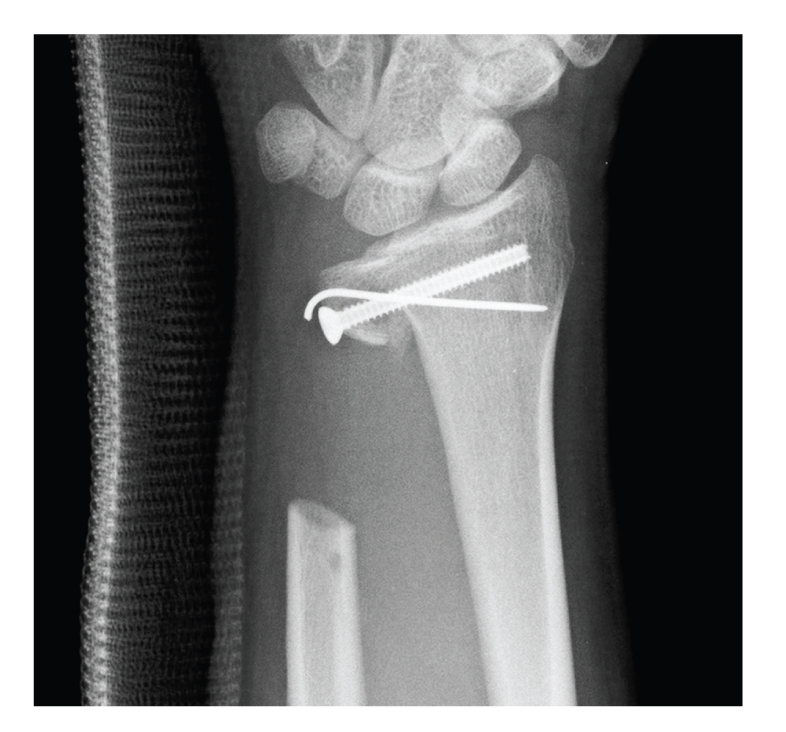
Iliac bone was grafted to the sigmoid notch of the radius. The grafted iliac bone was fixed with a small cortical screw and a 1.5 mm diameter Kirschner wire.

 Reconstructive surgery with tumor resection was performed under general anesthesia six weeks after his first visit to our hospital. The distal ulna including healthy proximal bone was resected en bloc to preserve the origin at the ulnar fovea of the triangular fibrocartilage with the ulnar collateral ligament. Iliac bone was harvested from the contralateral iliac crest by using separate instruments and was grafted to the ulnar side of the sigmoid notch of the radius-like Sauvé-Kapandji procedure. The grafted iliac bone was fixed with a small cannulated cortical screw and a 1.5 mm diameter Kirschner wire (Figure 3). The triangular fibrocartilage with the ulnar collateral ligament, which had been preserved, was attached to the distal radial aspect of the grafted iliac bone in order to reconstruct the ulnar support.

 Thereafter, we performed stabilization procedure of the proximal stump of the ulna as described previously [4–6]. The cut end of the proximal stump of the ulna was smoothed with a rongeur and rasp. A 3.5 mm hole was drilled from the dorsoulnar aspect of the ulnar shaft into the intramedullary cavity. The extensor carpi ulnaris tendon was split in the central sulcus and the radial half was released at the ulnocarpal level. It was then reflected proximally, leaving it attached at the musculotendinous junction. This proximally based slip, approximately six to eight cm long, was then passed into the medullary canal through the drill hole, retrieved at the distal stump of the ulna, and then sutured back on itself in an interlacing fashion. Final pathological examination confirmed a benign giant-cell tumor (Grade II).

 A long arm splint was applied for two weeks, after which gentle active motion of the wrist and forearm was encouraged. Bony fusion between the radius and grafted ilium was confirmed on X-ray films eight weeks after the operation. Full range of motion in all directions was allowed and encouraged.

**Figure 4:**
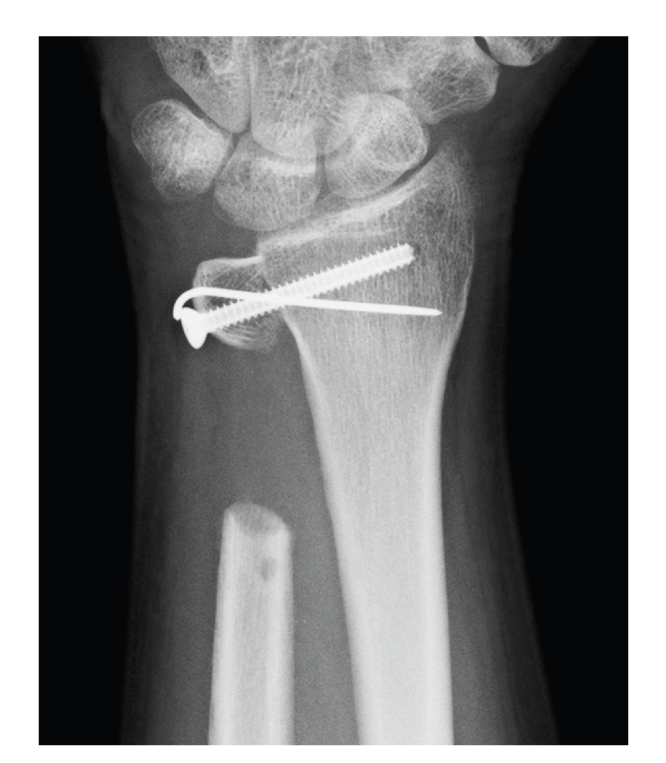
One year after the operation, X-ray showed no recurrence of the tumor, no absorption of the grafted bone, and no ulnar translation of the entire carpus. There was no convergence of the proximal stump of the ulna toward the radius.

 The patient's wrist was free from pain one year after the operation, the range of motion of the wrist was full (90° in dorsiflexion, 80° in volar flexion, 90° in pronation, and 90° in supination), and the grip strength was improved to 37 kgf. The stability of the proximal stump of the distal ulna was also maintained. X-rays of the patient's left wrist showed no recurrence of the tumor, no absorption of the grafted bone, and no ulnar translation of the entire carpus. There was no convergence of the proximal stump of the ulna toward the radius (Figure 4). Chest X-ray showed no metastases in the lung. 

## 3. Discussion

 Giant-cell tumor (GCT) of bone is a rare and essentially benign tumor [1]. GCT occurred in the ulnar epiphysis is furthermore extremely rare [2]. There has been an intralesional curettage with bone or artificial material graft or en bloc resection in the treatment of a low-grade GCT of the distal ulnar end. Resection of the distal end of the ulna, so-called the Darrach's operation, is a simple and easy solution but long-term results are still unpredictable, especially with high stress manual labor [7–9]. This may be attributed to abnormal stress distribution after resection of the distal ulna. 

 Resection of the distal ulnar end is most commonly performed for disorders of the distal radioulnar joint [10–12]. Long-term results of the Darrach's procedure are not predictable [13]. Ulnar carpal translation and snapping of the distal ulnar end and finger extensor tendon rupture due to instability of the ulna may occur as in our case [8]. The Sauvé-Kapandji procedure [4–6, 14] and radiolunate fusion have been used for reconstruction to prevent ulnar translation of the entire carpus [15]. However, there are many problems including the necessity of a healthy distal end of the ulna and an unacceptable decrease in range of motion of the wrist after these surgeries.

 Goal for our young active patient was to preserve as much wrist instability and movement as possible. Hashizume et al. [16] used a longer iliac bone graft to achieve the buttress effect against axial stress loading, hence the term “ulnar buttress arthroplasty”. Our method should be called a modification procedure described by them.

 Excessive resection of the distal ulna usually results in painful instability of the stump or a click [17–20]. In the present case, the distal stump of the ulna after resection of the distal ulna including GCT lesion was stabilized by the method previously reported in our papers [5, 6].

 In conclusion, our procedure is useful for the ulnar support reconstruction after wide resection of the distal ulna including ulnar head. The ulnar support consisting of iliac bone graft, preservation of the TFCC and the ulnar collateral ligament, can lead to good results.
